# Supplementary material for: Essential functions of Runx/Cbfβ in gut conventional dendritic cells for priming Rorγt+ T cells
Source: Life Sci Alliance. 2019 Dec 9;3(1):e201900441. doi: 10.26508/lsa.201900441 (PMC6907387; doi:10.26508/lsa.201900441)
Supplement: Supplementary file 1 [file LSA-2019-00441_TableS1.doc]

Supplemental Table 1

| Score | Histological Change |
| --- | --- |
| 0 | There were no changes observed. |
| 1 | Minimal scattered mucosal inflammatory cell infiltrates, with or without minimal epithelial hyperplasia. |
| 2 | Mild scattered to diffuse inflammatory cell infiltrates, sometimes extending into the submucosa and associated with erosions, with minimal to mild epithelial hyperplasia and minimal to mild mucin depletion from goblet cells. |
| 3 | Mild to moderate inflammatory cell infiltrates that were sometimes transmural, often associated with ulceration, with moderate epithelial hyperplasia and mucin depletion. |
| 4 | Marked inflammatory cell infiltrates that were often transmural and associated with ulceration, with marked epithelial hyperplasia and mucin depletion. |
| 5 | Marked transmural inflammation with severe ulceration and loss of intestinal glands. |
